# Optimization of Mass Flow in the Synthesis of Ferromagnetic Carbon Nanotubes in Chemical Vapor Deposition System

**DOI:** 10.3390/ma14030612

**Published:** 2021-01-28

**Authors:** Grzegorz Raniszewski, Łukasz Pietrzak

**Affiliations:** Institute of Mechatronics and Information Systems, Faculty of Electrical, Electronic, Computer and Control Engineering, Lodz University of Technology, Stefanowskiego 18/22 str., 90-924 Lodz, Poland; lukasz.pietrzak@p.lodz.pl

**Keywords:** carbon nanotubes, chemical vapor deposition, ferromagnetic nanocontainers

## Abstract

Carbon nanotubes have unique properties, which make it possible to be applied in a variety of sensing applications. Moreover, by controlling the synthesis chemistry process, it is possible for carbon nanotubes to either fill or attach to its surface metal particles, e.g., iron. In an industrial scale, the yield and purity of the final product is very important. This work describes the chemical vapor deposition (CVD) method of carbon iron nanocontainers with maximum nanoparticles to impurities ratio. As one of the main parameters, the mass flow of gases was considered. To investigate the quality of the product, the scanning electron microscopy and thermogravimetric methods were used. Results for different process conditions were presented and discussed. The low gas velocity and high temperatures may affect the catalyst decomposition and ionization. The optimum flow and temperature in the reactor were determined.

## 1. Introduction

Carbon nanotubes are an object of interest for many researchers in many fields such as sensing applications [1,2,3], automotive [4], computer science [5,6], medical diagnosis and therapy [7,8,9,10], chemical industry [11], and material engineering [12,13,14]. Carbon nanotubes have unique properties such as high thermal and electrical conductivity [15], an aspect ratio which exceeds 1:1000, high flexibility, very high tensile strength [16], high thermal stability [17], and chemical resistance [18]. Due to its properties, carbon nanotubes can be applied in many industries such as in electronics, e.g., field-effect transistors (FETs) [19,20], as an electronic material for next-generation electronic devices [21,22,23,24], even in CNT computers [25,26].

Carbon nanotubes were applied in a textile industry [27], as elements of supercapacitors [28], as field emitters [29,30], and as an element of composite polymers [31]. Moreover, carbon nanotubes have a high potential as drug carriers [32,33,34]. In the literature, we can find attempts of application in thermal tumor cells ablation [35,36]. Any application of carbon nanotubes requires a high-yield, repetitive production process. The final product has to be characterized by specified, desired properties.

The structure of carbon nanotubes are divided into two groups—multi-walled carbon nanotubes (MWCNTs) and single-walled carbon nanotubes (SWCNTs). The most common method of carbon nanotubes synthesis is the arc discharge method, laser ablation, and chemical vapor deposition (CVD).

Carbon nanotubes filled with a ferromagnetic material may lead to an increase in the capacity of magnetic read-write devices. The nanoparticle size can represent a single domain and the carbon nanotube walls are nonmagnetic separating regions between the nanoparticles [37].

One of the most common areas of ferromagnetic carbon nanotubes application is that individual nanotubes can be used as cantilever tips in magnetic force microscopy (MFM). These nanotubes can be produced by the selective growth of aligned Fe-filled multi-walled nanotubes [38]. Ferromagnetic-filled carbon nanotubes are very interesting for applications in biomedicine for imaging, diagnosis, and therapy [39].

In this work, a catalytic chemical vapor deposition (CCVD), as an efficient and low-cost method for mass production, is described.

## 2. Research Set-Up

The catalytic chemical vapor deposition is one of the methods of carbon nanotubes synthesis. In [40], a swirled floating catalyst technique with a xylene/ferrocene as the catalyst was described. There are also attempts to obtain single-walled nanotubes but the final product was not aligned [41,42]. In thermal ablation, multi-walled carbon nanotubes are more convenient for functionalization, targeting, and filling. Single-walled carbon nanotubes, due to their less damaged structure, are difficult for further modification. In the research, a catalyst is injected into the system as a liquid solution. In the literature, the process can be seen as a liquid source chemical vapor deposition (LSCVD). The synthesis of ferromagnetic CNTs employs a limited group of catalysts—mostly metalorganic compounds with a general formula Me–(C_5_H_5_)_2_, where Me means metal from the transition metals group. Ferrocene Fe(C_5_H_5_)_2_ (Sigma-Aldrich) was used as a solution in xylene (Chempur) in the analytical reagent (AR) grade. The role of xylene is not only to work as a solvent but also to be an additional source of carbon elements. The main criterium for the method selection was the possibility to control the process by an easy change of parameters such as the catalyst dosing rate, temperatures in the zone of catalyst vaporization, temperatures in the zone of carbon material deposition, flow rate of gases, and time of synthesis. The proper adjustment of parameters results in the perpendicular formation of aligned carbon nanotubes. In cases with catalysts in solid states, the amount of sublimated material is limited, thus these processes are periodical. If the catalyst is injected in a continuous way, it results in the continuous formation of ferromagnetic CNTs. This process enables obtaining a relatively large amount of material during the process.

Although mathematical models for iron particles formation and the kinetic model of size control of the generated nanoclusters can be found [43,44,45], we focused on the synthesis of MWCNTs with the maximum iron filling. The aim of this work is to determine a suitable temperature of ferrocene solution vaporization, the rate of catalyst dosing, flow rate of gases, and temperatures in the deposition zone. As a criterium of success, the yield and purity of MWCNTs were established. Carbon nanotubes should form an aligned, perpendicular to the surface forest-like structure and be homogeneous over the entire surface of the substrate. The essential aspect of mass flow selection and gas composition is the final product with a possible minimum amount of impurities such as amorphous carbon, graphite, etc.

## 3. The Experiment

The catalytic chemical vapor deposition to obtain ferromagnetic multi-walled carbon nanotubes was used. The system was composed of:

- The source of gases;
- Flowmeters with regulators;
- Injection pump;
- Tube furnace;
- Gas exhaust system.

**Figure 1:**
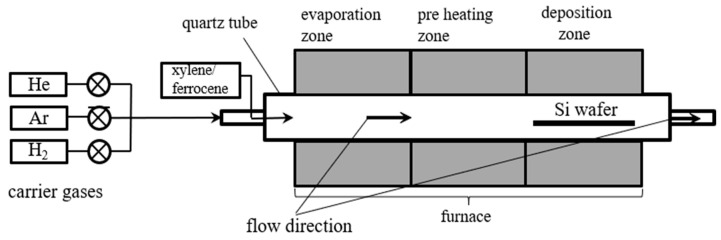
Catalytic chemical vapor deposition (CCVD) system for ferromagnetic carbon nanotubes synthesis.

The Linde gas cylinders were applied and argon and helium with a purity of 99.9% were used. Hydrogen was produced in the electrolysis process in a Linde HiQ H2 FID hydrogen carrier gas generator. Dosing valves and flowmeters were used to regulate the mass flow of gases. Injection pump Medima S2 was employed to dose the catalyst solution. The tube furnace was divided into three zones. Each zone could be regulated independently in the temperature range up to 1050 °C. The first zone was responsible for the preheating of gases and catalyst vaporization. In the second zone, neutral gases and iron particles were heated to the adjusted temperature. The last zone was the deposition zone where carbon nanotubes were formed and deposited on the substrate. As a substrate, a silicon wafer was used. As an exhaust gas system, a barbotage column and ventilation pump were used. Figure 1 shows the scheme of the process.

The process started with the silicon stripe placement inside the quartz tube in the third zone. The quartz tube with an inner diameter of 30 mm and length of 1200 mm was placed inside the tube furnace, connected to the injection pump on one side, and to the exhaust gas purification system on the other side. Then, the quartz tube was tightly sealed. Air was removed from the reactor by a 10 min ventilation by argon and helium to remove the air present in the quartz tube and prevent the oxidation of synthesis products. The argon flow velocity as well as helium was set to 1 standard liter per minute each. The vaporization zone was 300 °C, the preheating zone was 450 °C, and the reaction zone was 850 °C. In the vaporization zone, the temperature guarantees the vaporization of the substrate. In this zone, the dynamics of vaporization can be controlled, thereby the amount of iron and carbon particles in the reactor. The preheating zone is responsible for keeping the catalyst in the gaseous form. In the reaction zone, carbon nanotubes are formed. The important factor for carbon nanotubes formation is a stable temperature. Heat fluctuations may lead to the damage of carbon nanotubes structure and in effect its destruction. Furthermore, carbon nanotubes in variable conditions may be closed, which prevent iron encapsulation. Therefore, the temperature accuracy was set to ±2 °C. Next, the helium inflow was stopped and the argon flow velocity was reduced to 0.5 SLPM. The hydrogen was introduced to eliminate oxygen residues from the substrate surface (velocity flow 0.08 SLPM). The solution of ferrocene in xylene was introduced. The rate of injection was set to 9.5 ml/min. The process lasted 90 min and the hydrogen inflow was stopped afterward. The temperature of the tube furnace was subsequently decreased to the temperature below 50 °C to avoid oxidation in the case of contact of the hot nanoparticles with air.

Carbon nanotubes were synthesized by the vaporization and decomposition of liquid hydrocarbon xylene C_8_H_10_ in a mixture with volatile ferrocene Fe(C_2_H_5_)_2_ as a catalyst at an atmospheric pressure (concentration of ferrocene 0.02 g/ml). Argon was used as a carrier gas on the surface of the silicon substrate. The tube furnace was specially designed for these purposes.

## 4. Measurements

The samples were dispersed in the organic solvent (Chempur Poland dichloroethane AR grade) in the ultrasonic homogenizer. The scanning electron microscopy SEM In-Touch-Scope™ JSM-IT200 (JEOL Ltd., Tokyo, Japan), was used to analyze the samples topography. Energy-dispersive X-ray spectroscopy was prepared by detecting characteristic X-rays generated from a specimen by the Jeol In-Touch-Scope™ JSM-IT200, scanning electron microscope. The thermogravimetric analysis (TGA) in the air (sample heating—10 °C/min) was carried out to determine the iron particles concentration in the final product (TA Instruments 2950 TGA HR, DE, USA). The temperature distribution inside the reactor was calculated by the finite element method in FEMM 4.2 (USA). Due to the long synthesis time, it was assumed that the reactor is in a static state. It was based on the thermal conductivity specific for the used materials. The constant temperature of the heaters was assumed for every furnace zone.

## 5. Discussion

In order to determine the optimum temperature of the synthesis, a series of measurements were conducted. The selected results are presented in Table 1.

Samples were weighed, thermogravimetrically analyzed, and characterized by SEM microscopy. Based on the obtained results, it was possible to indicate clue parameters for the process and to determine the influence of the mass flow of gases on the final product. The most advantageous conditions appeared in sample no. 4 and the results are summarized in Table 2. For sample no. 1 (the highest flow rate), the yield was too low. The carbon nanotubes growth rate was 0.01 to 0.03 mg/cm^2^/h. The reduction of the flow rate resulted in a slight yield increase to 0.05 mg/cm^2^/h (sample no. 2). The yield of 0.1–0.3 mg/cm^2^/h also seems to be insufficient (sample no. 3). A further reduction of the flow rate to 0.75 SLPM (Ar/H_2_ 0.675:0.075) resulted in a maximum growth equal to 0.7–1.2 mg/cm^2^/h (sample no. 4). Flow rates below 0.5 SLPM gave smaller amounts of nanotubes. The results of the experiment are shown in Table 2.

**Figure 2:**
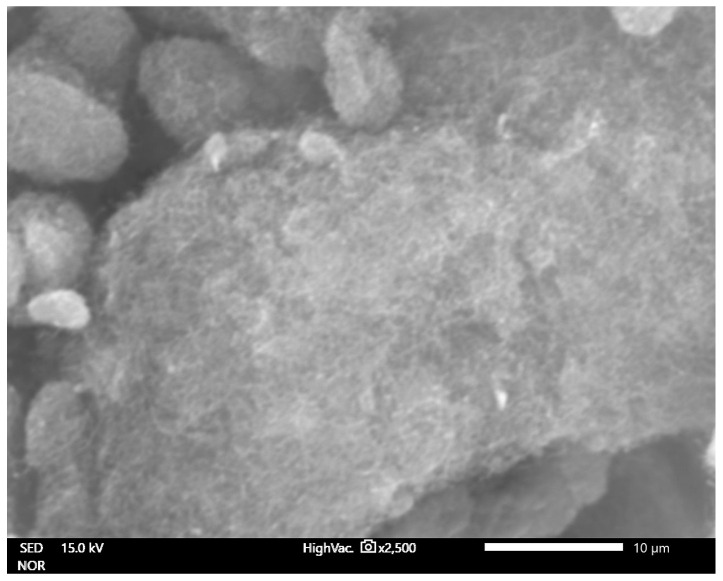
SEM image of ferromagnetic multi-walled carbon nanotubes (MWCNTs)—sample no. 2 (high flow velocity).

**Figure 3:**
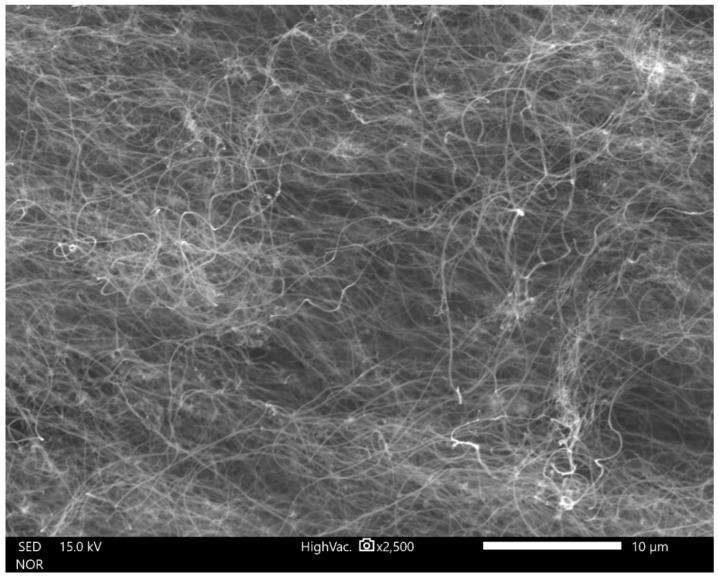
SEM image of ferromagnetic MWCNTs—sample no. 4 (optimum flow velocity).

**Figure 4:**
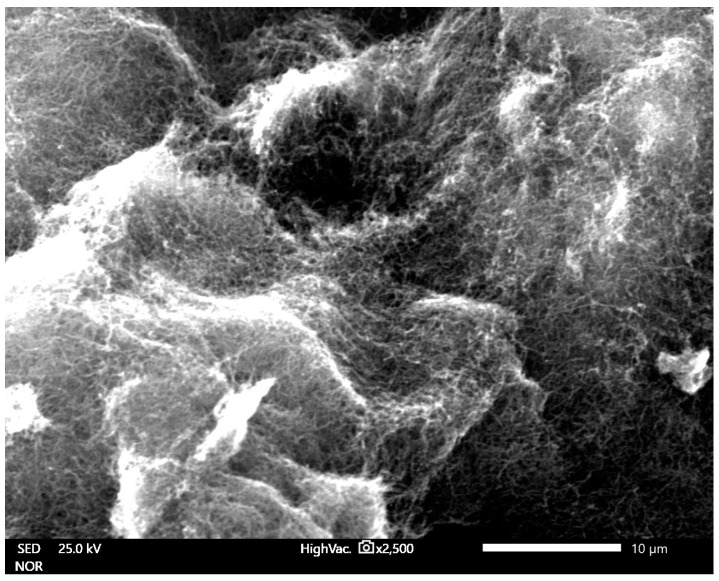
SEM image of ferromagnetic MWCNTs—sample no. 5 (low flow velocity).

The maximum growth rate was noticed at a temperature of 800 °C for all the samples (all conditions). An estimation of the optimum flow rate enabled the production of the carbon nanotubes in a quantity sufficient for further characterization. Figure 2, Figure 3 and Figure 4 show the carbon nanotubes from different synthesis conditions.

According to the conducted SEM analysis, the dependence of the final product quality on the mass flow can be observed. For high fluxes, short, tangled carbon structures are dominant. There are no formations indicating the presence of iron. In low velocities, MWCNTs are visible but still finding the best conditions for the best product is required (Figure 3).

The electron microscope equipped with a Roentgen spectrometer (EDS) JEOL JED-2300 (Japan), allowed determining the chemical composition of the tested samples.

**Figure 5:**
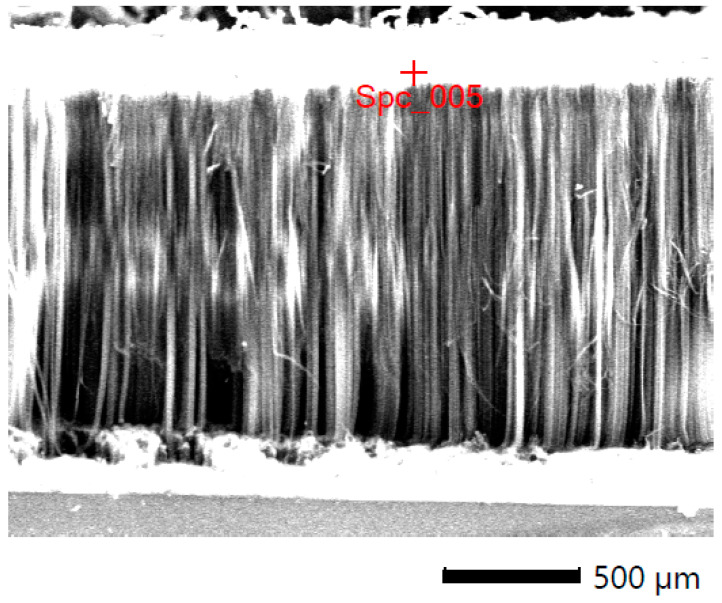
SEM image of ferromagnetic MWCNTs—sample with an optimum flow velocity. Side view of the aligned nanotubes.

**Figure 6:**
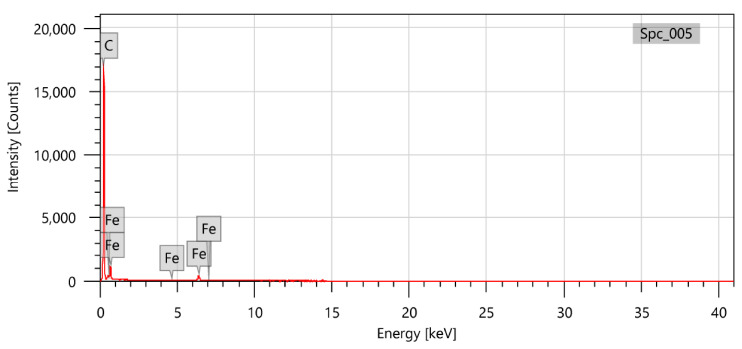
EDS analysis of the sample with an optimum flow velocity. Side view of the aligned nanotubes.

Figure 5 shows a side view of the carbon nanotubes aligned perpendicularly to the base (silica wafer). Figure 6 is the high magnification of the side of a carpet showing entangled carbon nanotubes, which is the common state of raw material from the synthesis. The length of the carbon nanotube forest may exceed 1 mm. In the literature, we can find attempts to synthesize the aligned carbon nanotubes with nickel/ferrocene-hybridized and ferrocene catalysts [41], but in the compared cases, the carbon nanotubes forest was up to a few hundred micrometers in height.

Moreover, the chemical composition of the samples was tested in the middle of the carpet height and on the top. Both examinations resulted in showing the carbon and iron atoms in the structure of the nanotubes. For this sample, the EDS resulted in Fe contamination at the level of 9.74 ± 0.18 wt%. Furthermore, a higher content is obtained on the top of the carbon nanotubes, which indicates a “tip up” growth mechanism. The examination also proves the iron content in synthesized carbon nanotubes, which is the basis of the medical application of the obtained material.

**Figure 7:**
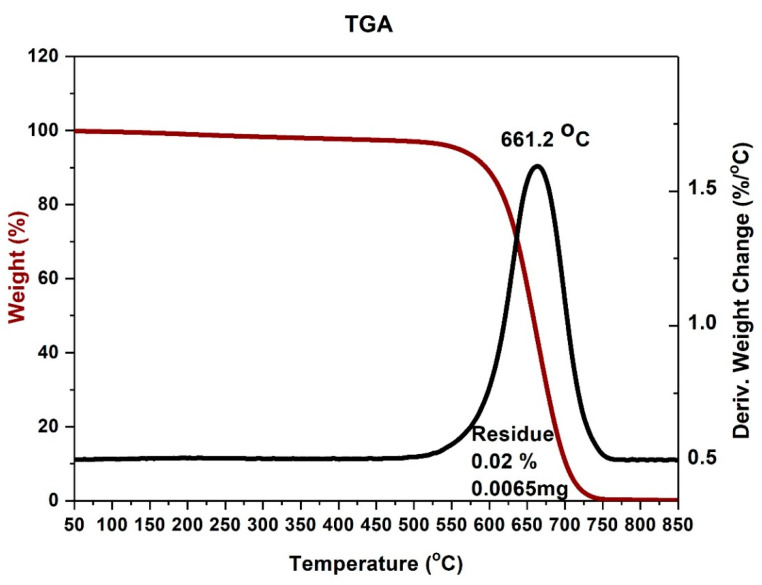
TGA of ferromagnetic MWCNTs—sample no. 2 (high flow velocity).

**Figure 8:**
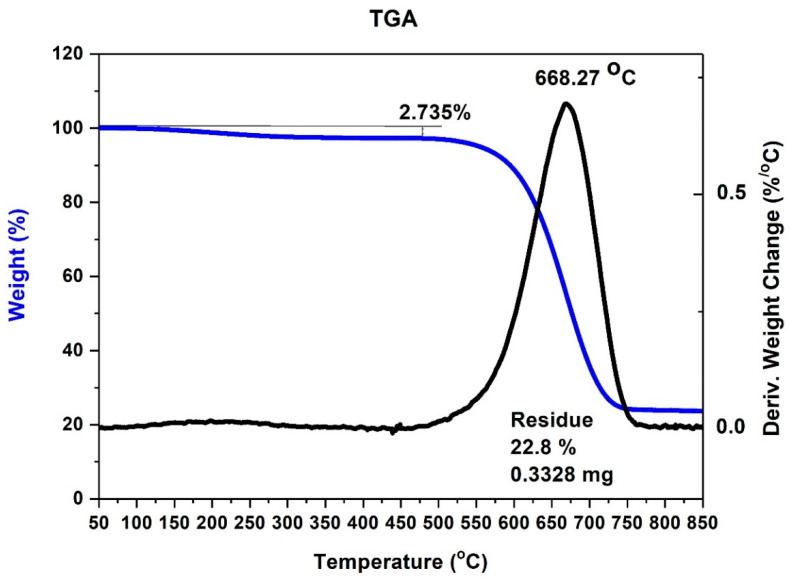
TGA of ferromagnetic MWCNTs—sample no. 4 (optimum flow velocity).

To calculate the purity of the product, an additional thermogravimetric analysis was conducted (Figure 7 and Figure 8).

The thermogravimetric analysis proved that high gas velocities limit the connections of iron particles and ions to the carbon nanotubes surface. The residue is much smaller than 1%. It means that almost all the material is decomposed at a temperature over 725 °C (Figure 7). The long residence time is a result of low velocities. A longer time enables ferrocene decomposition and possibly ionization.

**Figure 9:**
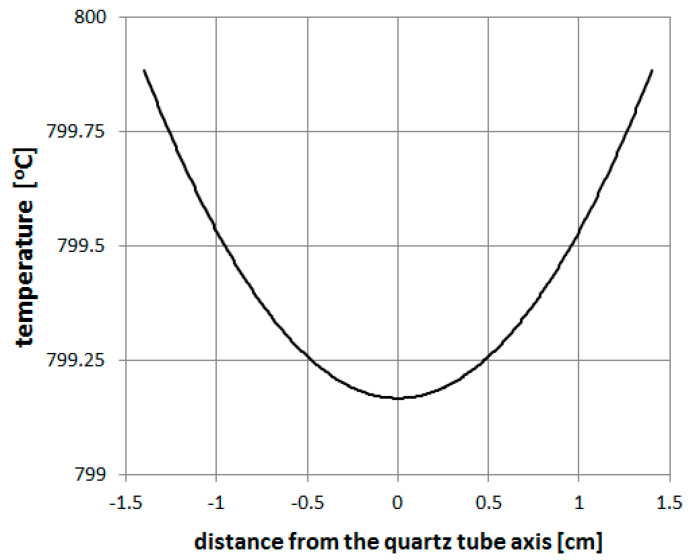
Temperature distribution in the quartz tube cross-section in the reaction zone.

To calculate the temperature distribution in the quartz tube in the reaction zone, a FEMM software was used. In Figure 9, it can be seen that in the case of a small diameter of a quartz tube (<30 mm) the difference between the wall temperature and temperature in the tube axis does not exceed 1 °C.

The temperature distribution analysis enabled determining the substrate placement in the quartz tube and obtaining a homogenous material with proper filling with a ferromagnetic material.

## 6. Conclusions

In the experiment, a large variety of conditions were analyzed. The authors focused on the carried gas flow rate change. SEM images and the thermogravimetric analysis indicated a relationship between the growth rate of ferromagnetic MWCNTs and temperature. The test carried out under different conditions determined the synthesis parameters for the highest possible yield. Wherein, the yield costs of gas and catalysts were omitted. An optimum parameter, with 300 °C in the vaporization zone, 800 °C in the deposition zone, and an Ar/H_2_ flow rate equal to 0.5/0.08 SLPM, corresponds to a flow velocity of 2 cm/s in the 28 mm quartz tube. It may be connected with a required time for ferrocene decomposition. Gas-phase metallocenes such as ferrocene have a low ionization potential, very low solvation energies, and slow proton transfer with a small temperature coefficient [46,47]. Although the ionization rate does not exceed 0.1%, the presence of iron atoms and ions is enough to initiate an ion=molecule reaction and the carbon nanotubes growth. During the 90 min synthesis, it was possible to obtain over 10 mg of ferromagnetic nanotubes per sqcm. Future work will be focused on the maximum filling of MWCNTs. Ferromagnetic nanotubes from this system were used as nano heaters in the electromagnetic field and after chemical functionalization to the thermal ablation of cancer cells [48,49].

## Figures and Tables

**Table 1:** Conditions of the experiments.

Sample No.	Temp. in the Zone 1 [°C]	Temp. in the Zone 2 [°C]	Temp. in the Zone 3 [°C]	Concentration of Ferrocene [g/L]	Ar/H_2_ Flow Rate [SLPM]	Solution Injection Rate [mL/h]
1	300	450	750–850	2	2/0.4	9
2	300	450	750–850	2	0.8/0.2	9
3	300	450	750–850	2	0.675/0.075	9
4	300	450	750–850	2	0.5/0.08	9
5	300	450	750–850	2	0.380/0.060	9

**Table 2:** Results for different flow velocities.

Sample No.	Ar/H_2_ Flow Rate	Flow Velocity	Growth Rate
	[SLPM]	[cm/s]	[mg/cm^2^/h]
1	2/0.4	8.8	0.01−0.03
2	0.8/0.2	3.5	0.05−0.06
3	0.675/0.075	2.5	0.1−0.3
4	0.5/0.08	2.0	0.7−1.2
5	0.380/0.060	1.5	0.2−0.5

## Data Availability

Data Sharing is not applicable.
